# YTH domain family: potential prognostic targets and immune-associated biomarkers in hepatocellular carcinoma

**DOI:** 10.18632/aging.203674

**Published:** 2021-11-08

**Authors:** Miaomiao Liu, Zijin Zhao, Yuan Cai, Peng Bi, Qiuju Liang, Yuanliang Yan, Zhijie Xu

**Affiliations:** 1 Department of Nuclear Medicine (PET-CT Central), Xiangya Hospital, Central South University, Changsha, China; 2 Department of Neurosurgery, Xiangya Hospital, Central South University, Changsha, China; 3 Department of Pathology, Xiangya Hospital, Central South University, Changsha, China; 4 Department of Pharmacy, Xiangya Hospital, Central South University, Changsha, China; 5 National Clinical Research Center for Geriatric Disorders, Xiangya Hospital, Central South University, Changsha, China

**Keywords:** hepatocellular carcinoma, YTH domain family, expression profiles, infiltrating immune cells, methylation

## Abstract

Hepatocellular carcinoma (HCC) is the most common high malignancy with insidious onset, invasive fast-growing, high recurrence rate and fatality. YTH domain family plays essential roles in development of HCC. However, the biological function of YTH domain family in HCC have not been clarified. Here, through evaluating the expression profiles of YTH domain family, we found that upregulated YTHDF1 might be more significant and valuable in development and progression of HCC. There was a strong correlation between YTHDC1, YTHDF1 and YTHDF2 and pathological stage of HCC patients. Kaplan-Meier plotter revealed that HCC patients with high level of YTHDF1 and YTHDF2 were highly related to a shorter overall survival time, and low level of YTHDF1 (p = 0.0017) has an important association with a longer progression-free survival time. Genetic alterations using cBioPortal revealed that the alteration rates of YTHDF3 were the highest. We also found that the functions of YTH domain family were linked to several cancer-associated pathways, including peptidyl-serine modification, peptidyl-tyrosine modification and negative regulation of cellular component movement. TIMER database indicated that the YTH domain family had a strong relationship with the infiltration of six types of immune cells (macrophages, neutrophils, CD8+ T-cells, B-cells, CD4+ T-cells and dendritic cells). Next, Ualcan databases revealed that the global methylation levels of YTHDC1 was higher in HCC patients, while YTHDF2 was lower in HCC patients. In conclusion, our findings will enhance the understanding of YTH domain family in HCC pathology, and provide novel insights into YTH-targeted therapy for HCC patients.

## INTRODUCTION

Hepatocellular carcinoma (HCC) has become one of the most common and aggressive malignant disease to bring about worldwide concern, which ranks as the sixth of morbidity and the third of mortality in all malignant tumors [1, 2]. The onset of this malignant disease is usually indetectable and insidious resulting in having developed into middle and advanced stage [3]. The therapeutic outcome of HCC is unfavorable leading to the 5 years survival rate of HCC is less than 19% [4]. Consequently, exploration of novel molecular markers will play an essential role for improving the prognosis of HCC patients.

Accumulating statistics indicate that among a series of RNA modification adenosines, N6-methyladenosine (m6A) plays the most significant part in modulating numerous cellular processes including cancer biological functions [5, 6]. Meanwhile, a recent study revealed that m6A is linked to the pathogenesis and development of HCC by regulating several targeted genes [7]. The YT521-B homology (YTH) domain family members, including YTHDC1, YTHDC2, YTHDF1, YTHDF2 and YTHDF3, are the main "m6A readers" [8]. Furthermore, YTH domain proteins are mostly enrolled in the tumorigenesis and tumor progression [9, 10]. However, the specific mechanisms of YTH domain family members in HCC development and disease progression still require further confirmation and research.

Despite the YTH domain family plays significant role in cancer development, the systematic analysis of YTH domain family expression profiles and functions in HCC were still insufficient. In our study, we devoted to explore the expression features and potential biological functions of YTH domain family in HCC patients (Supplementary Table 1).

## RESULTS

### Aberrant expression of YTH domain family in HCC patients

**Figure 1:**
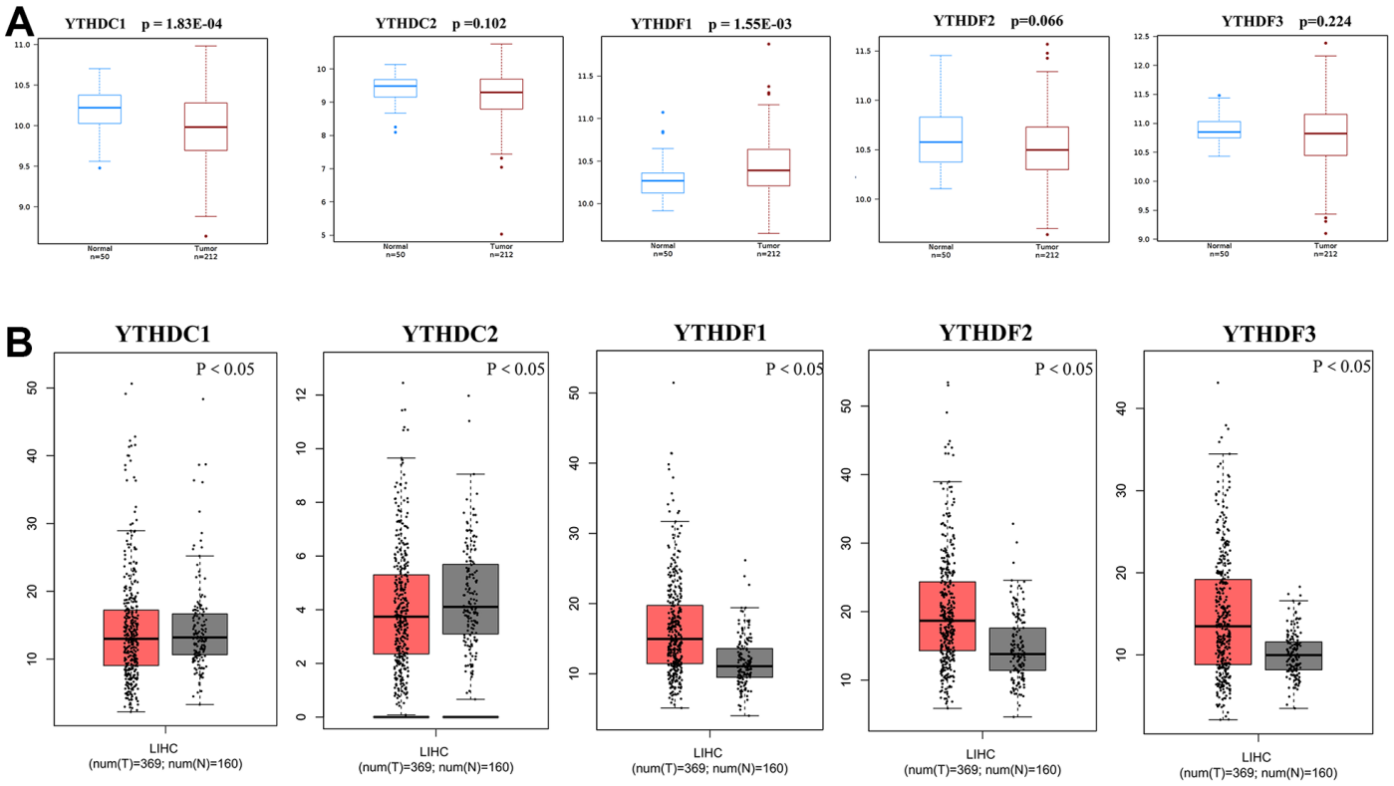
**Differential mRNA expression analysis of the YTH domain family in HCC and normal tissues.** (**A**) The expression profiles were collected from the Wanderer databases. (**B**) The expression profiles were collected from the GEPIA databases.

We used TIMER database to detect the expression levels of YTH domain family members. And the result showed that all of YTH domain family members were significantly raised in various malignant tumors (Supplementary Figure 1). Then, the mRNA expression levels of YTH domain family members in HCC tissues and normal tissues were evaluated by the Wanderer database. We found that the expression level of YTHDC1 (p = 1.83E-04) was significantly downregulated in HCC patients. While, the expression level of YTHDF1 (p = 1.55E-03) was higher in HCC tissues than in normal tissues (Figure 1A). Then, the GEPIA database was used to evaluate the gene expression of YTH domain family members in HCC tissues and normal tissues again. The analysis showed that expression levels of YTHDF1 (p < 0.05), YTHDF2 (p < 0.05) and YTHDF3 (p < 0.05) were significantly upregulated in HCC patients (Figure 1B). Further, the expression of YTH domain family members in HCC tissues and adjacent tissues were analyzed with HCCDB database. We found that the expression of YTHDC2 (p = 4.93E-04), YTHDF1 (p = 1.76E-26), YTHDF2 (p = 3.49E-07), and YTHDF3 (p = 3.21E-30) in HCC tissues were higher than in adjacent tissues (Table 1). Therefore, based on the above evaluation in these different databases, upregulated YTHDF1 might be more significant and valuable in development and progression of HCC.

**Table 1:** The expression of YTH domain family members in HCC tissues and adjacent tissues from the HCCDB database.

**Dataset**	**Gene**	**Type**	**Nums**	**Mean**	**STD**	**P-value**	**IQR**
HCCDB18	YTHDC1	HCC	212	3.103	0.3624	3.23E-01	0.4225
		Adjacent	177	3.069	0.3108		0.38
	YTHDC2	HCC	212	2.864	0.479	4.93E-04	0.5525
		Adjacent	177	2.713	0.3645		0.46
	YTHDF1	HCC	212	3.884	0.3942	1.76E-26	0.52
		Adjacent	177	3.447	0.3555		0.45
	YTHDF2	HCC	212	4.175	0.4425	3.49E-07	0.585
		Adjacent	177	3.972	0.3301		0.42
	YTHDF3	HCC	212	4.049	0.5341	3.21E-30	0.7025
		Adjacent	177	3.505	0.3027		0.31

**Figure 2:**
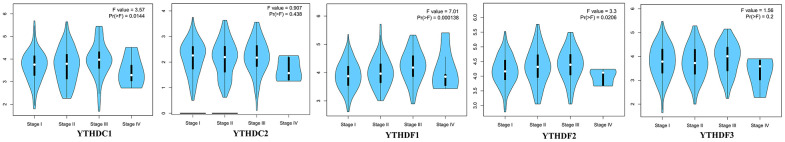
**The relationship between the expression of the YTH domain family and the pathological stage of HCC patients (GEPIA).** GEPIA databases were used to evaluate the correlations of the YTH domain family with the pathological stage of HCC patients.

Then, we explored the association between the expression profiles of YTH domain family and the pathological stages of HCC patients. As shown in Figure 2, YTHDC1 expression (p = 0.0144), YTHDF1 expression (p = 0.000138) and YTHDF2 expression (p = 0.0206) displayed a positive correlation with pathological stage. These data suggested that aberrant expression of YTH domain family members might participate in disease progression in HCC patients.

### The prognostic value of the YTH domain family in HCC patients

**Figure 3:**
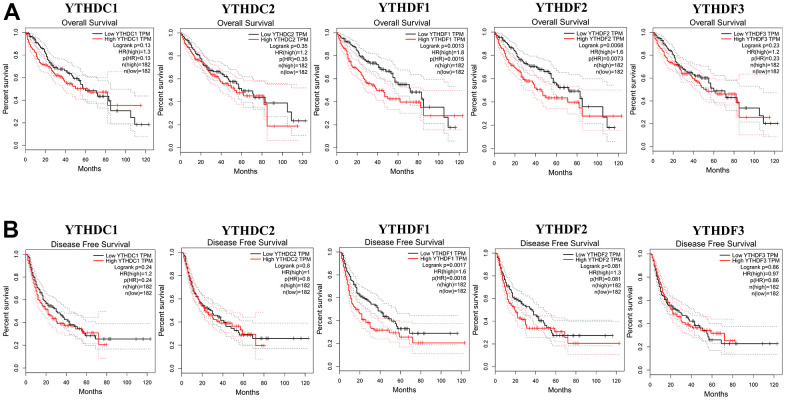
**The correlations of YTH domain family expression with OS and PFS in HCC patients.** Kaplan-Meier plotter was used to assess the correlation of YTH domain family members with the patients’ OS (**A**) and PFS time (**B**).

Then, The Kaplan-Meier plotter was used to evaluate the prognostic value of YTH domain family for patients with HCC. The data of overall survival (OS) curves are shown in Figure 3A. We found that a high transcriptional level of YTHDF1 (p = 0.0013) and YTHDF2 (p = 0.0068) were highly related to a shorter OS time. At the same time, we evaluated the prognostic significance of YTH domain family on the progression-free survival (PFS) of HCC patients. From Figure 3B, we know that low level of YTHDF1 (p = 0.0017) has an important association with a longer PFS time. Thus, the YTHDF1 might be more important and influential in prognostic value to HCC patients.

### Genetic alteration and functional enrichment analyses of YTH domain family in HCC patients

**Figure 4:**
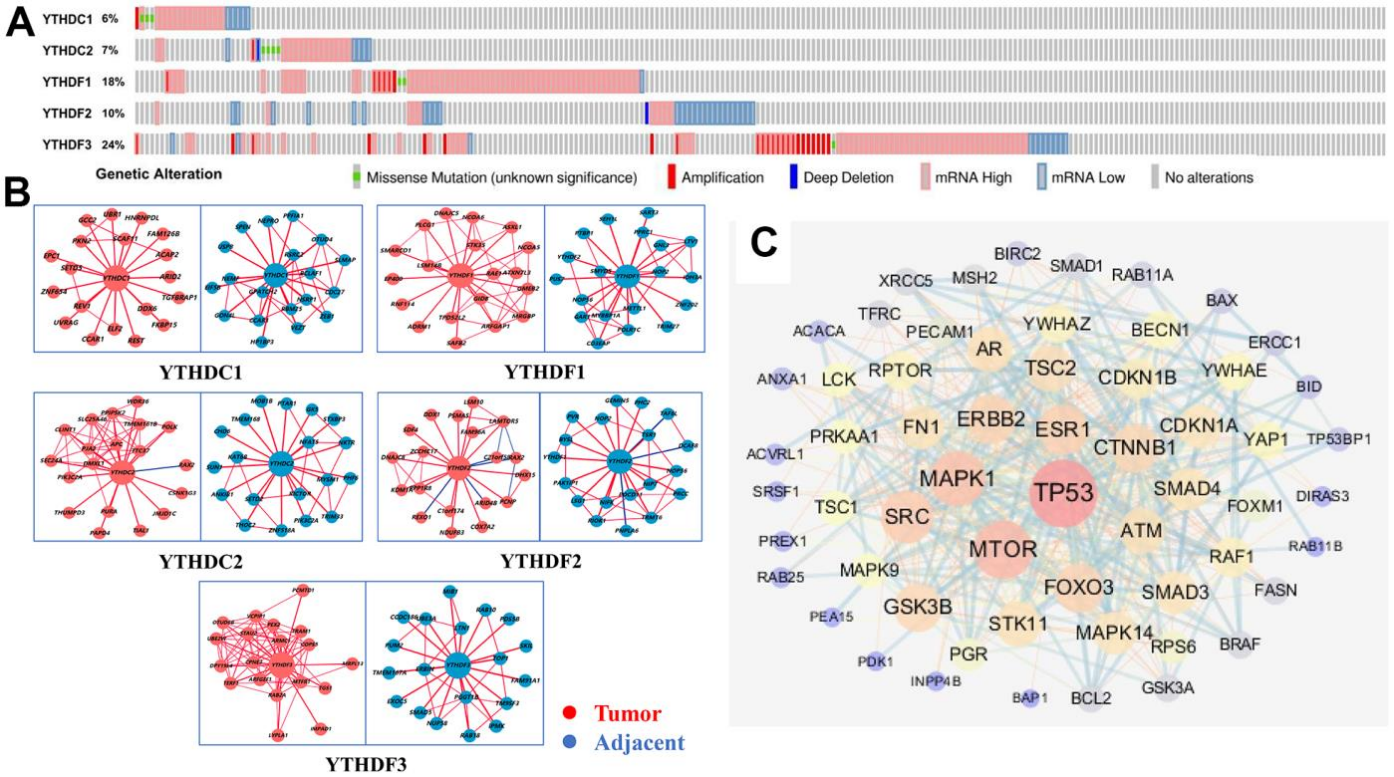
**Genetic alterations and interaction analyses of the YTH domain family in HCC patients.** (**A**) Genetic alteration of the YTH domain family in HCC patients analyzed with cBioPortal. (**B**) The HCCDB database was used to analyze the YTH domain family co-expressed genes in HCC and adjacent tissue samples. (**C**) The most frequently altered genes identified from cBioPortal that are linked to the YTH domain family in HCC patients.

To further analyze the alteration profiles of YTH domain members in patients with HCC, we conducted a series of surveys as follows. First, we used the cBioPortal database to evaluate the genetic alterations of YTH domain family. As shown in Figure 4A, we confirmed that the alternation rate of YTHDF3 was the highest in 24% of cases, whereas the lowest was for YTHDC1, which was only 6%. In addition, YTHDC2, YTHDF1 and YTHDF2 mutations occurred in 7%, 18% and 10% of the samples, respectively (Figure 4A).

Next, Using HCCDB database to evaluate the associated molecules of YTH domain family, we found that the interactive molecules of all members had altered significantly between tumor tissues and adjacent normal tissues, respectively (Figure 4B). Additionally, we used cBioPortal to extract 203 most frequently altered genes that were significantly linked to the YTH domain family in HCC patients (Supplementary Table 2). The data indicated that several hub genes, such as tumor protein p53 (TP53), mammalian target of rapamycin (MTOR) and mitogen-activated protein kinase 1 (MAPK1), were closely linked to the biological processes in YTH domain family in HCC patients (Figure 4C). Above data suggested that YTH domain family participated in the regulation of various signaling pathways involved in HCC biology.

**Figure 5:**
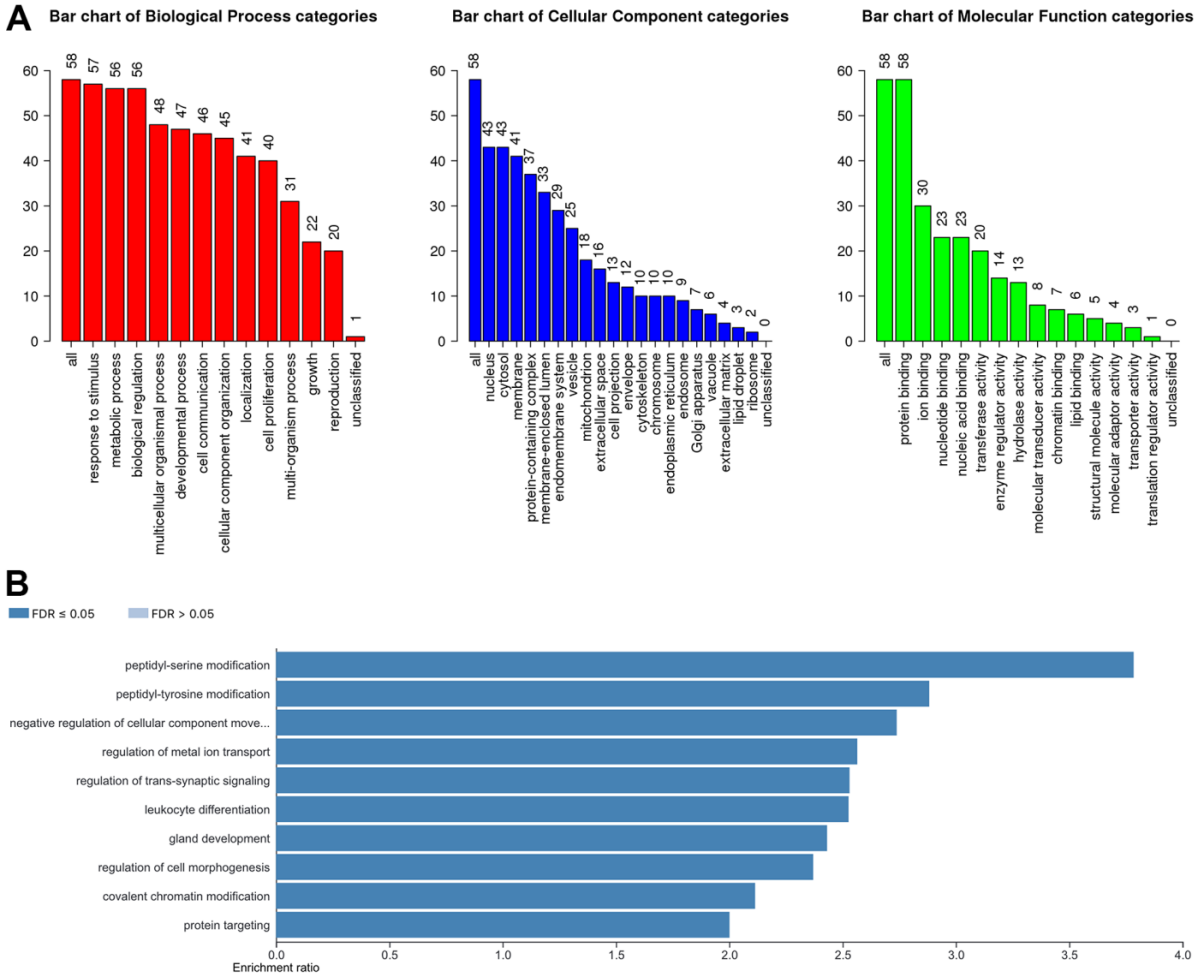
**The biological pathways of the YTH domain family were evaluated by the WebGestalt database.** (**A**) Bar plot of GO enrichment in cellular components, biological processes, and molecular functions. (**B**) The bar plot of KEGG enrichment.

In our study, we applied the WebGestalt database to evaluate the potential biological functions of YTH domain family in the occurrence and development of HCC. As shown in the Gene Ontology (GO) term analysis (Figure 5A), we know that the most highly enriched biological process (BP) category was response to stimulus, followed by metabolic process, biological regulation, etc. In the cellular component (CC) categories, membrane, nucleus, cytosol, membrane-enclosed lumen, protein-containing complex, endomembrane system and vesicle were highly accumulated. In the molecular function (MF) categories, the YTH domain family members were mainly enriched in protein binding, nucleotide binding, nucleic acid binding, ion binding and transferase activity. Meanwhile, the Kyoto Encyclopedia of Genes and Genomes (KEGG) pathway analysis results are shown in Figure 5B. We can conclude that several cancer-associated pathways, including peptidyl-tyrosine modification, peptidyl-serine modification and negative regulation of cellular component movement, were strongly linked to the potential biological functions of YTH domain family in the occurrence and development of HCC.

### The roles of YTH domain family on immune cell infiltration

**Figure 6:**
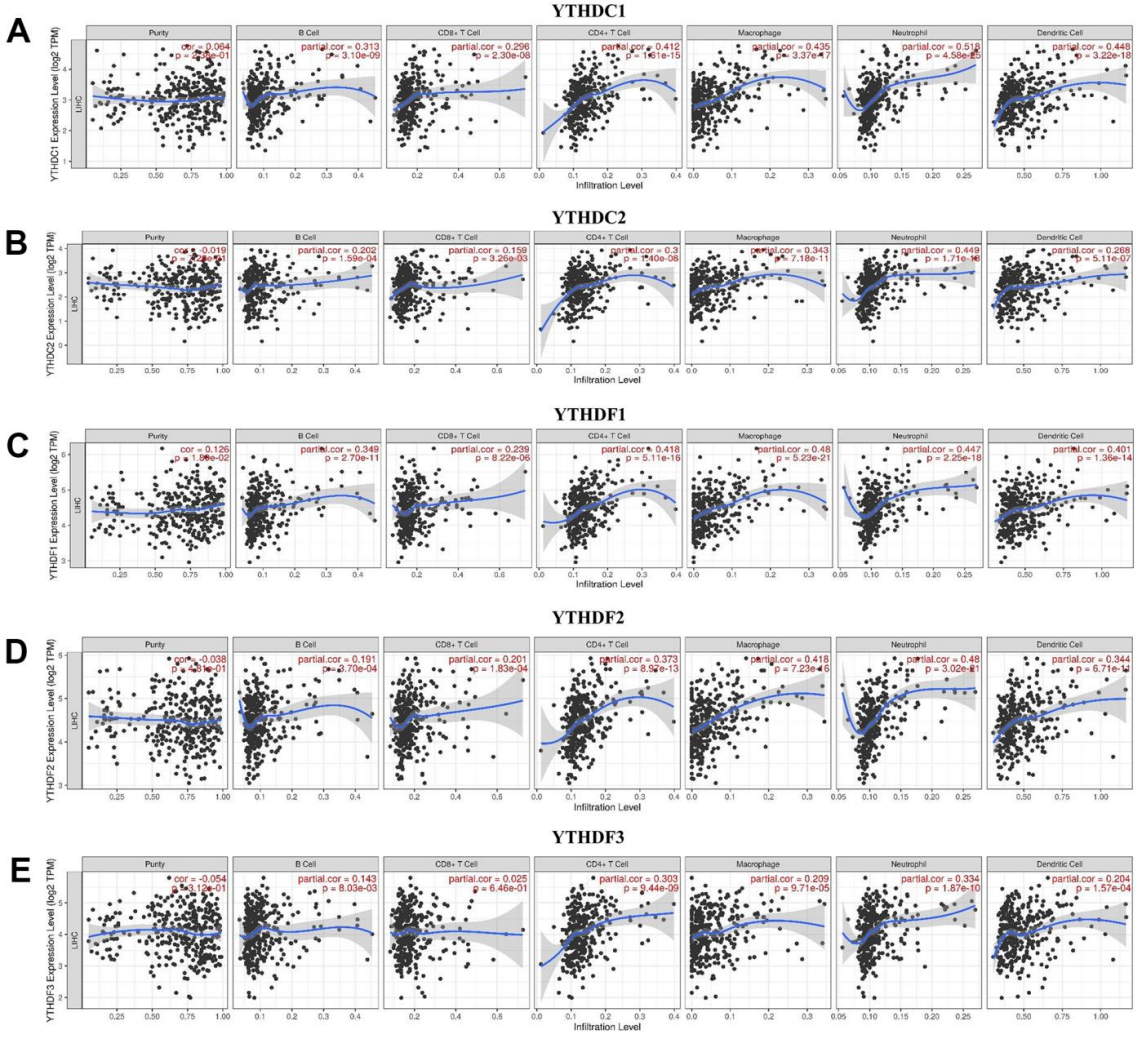
**The relationship between immune cell infiltration and the expression of the YTH domain family.** (**A**–**E**) The Timer database was used to analyze the effect of (**A**) YTHDC1, (**B**) YTHDC2, (**C**) YTHDF1, (**D**) YTHDF2, (**E**) YTHDF3 on the abundance of immune cells in HCC patients.

TIMER database was used to explore the relationship between immune cell infiltration and expression profiles of YTH domain family (Figure 6). The expression of YTHDC1 was positively linked to the infiltration of B cells (Cor = 0.313, p = 3.10e-09), CD8+ T cells (Cor = 0.296, p = 2.30e-08), CD4+ T cells (Cor = 0.412, p = 1.61e-15), macrophages (Cor = 0.435, p = 3.37e-17), neutrophils (Cor =0.518, p = 4.58e-25) and dendritic cells (Cor =0.448, p = 3.22e-18) (Figure 6A). There was a positive correlation between YTHDC2 expression and the infiltration of B cells (Cor = 0.202, p = 1.59e-04), CD8+ T cells (Cor = 0.159, p = 3.26e-03), CD4+ T cells (Cor = 0.300, p = 1.40e-08), macrophages (Cor = 0.343, p = 7.18e-11), neutrophils (Cor =0.449, p = 1.71e-18) and dendritic cells (Cor =0.268, p = 5.11e-07) (Figure 6B). YTHDF1 expression positively related with the infiltration of purity (Cor = 0.126, p = 1.80e-02), B cells (Cor = 0.349, p = 2.70e-11), CD8+ T cells (Cor = 0.239, p = 8.22e-06), CD4+ T cells (Cor = 0.418, p = 5.11e-16), macrophages (Cor = 0.480, p = 5.23e-21), neutrophils (Cor =0.447, p = 2.25e-18) and dendritic cells (Cor =0.401, p = 1.36e-14) (Figure 6C). We concluded that YTHDF2 expression was positively correlated with B cells (Cor = 0.191, p = 3.70e-04), CD8+ T cells (Cor = 0.201, p = 1.83e-04), CD4+ T cells (Cor = 0.373, p = 8.97e-13), macrophages (Cor = 0.418, p = 7.23e-16), neutrophils (Cor =0.480, p = 3.02e-21) and dendritic cells (Cor =0.344, p = 6.71e-11) (Figure 6D). We also found that the expression of YTHDF3 was positively related to the infiltration of B cells (Cor = 0.143, p = 8.03e-03), CD4+ T cells (Cor = 0.303, p = 9.44e-09), macrophages (Cor = 0.209, p = 9.71e-05), neutrophils (Cor =0.334, p = 1.87e-10) and dendritic cells (Cor =0.204, p = 1.57e-04) (Figure 6E). In addition, the Cox proportional hazard model revealed that B cells (p = 0.021), CD8+ T cells (p = 0.017), dendritic cells (p = 0.005) and YTHDF1 (p = 0.005) had a strong relationship with HCC patient prognosis (Table 2).

**Table 2:** The cox proportional hazard model of TYH domain family and six types of immune cells in HCC patients from timer database.

	**Coef**	**HR**	**95%CI_l**	**95%CI_u**	**P.value**	**Sig**
B_cell	-8.087	0.000	0.000	0.288	0.021	*
CD8_Tcell	-5.768	0.003	0.000	0.359	0.017	*
CD4_Tcell	-4.773	0.008	0.000	4.642	0.138	
Macrophage	3.593	36.356	0.228	5799.076	0.165	
Neutrophil	-0.241	0.786	0.000	75634.618	0.967	
Dendritic	5.056	157.029	4.755	5185.355	0.005	**
YTHDC1	-0.482	0.618	0.356	1.071	0.086	
YTHDC2	-0.217	0.805	0.542	1.194	0.281	
YTHDF1	0.763	2.145	1.258	3.659	0.005	**
YTHDF2	0.518	1.678	1.001	2.815	0.050	
YTHDF3	0.021	1.022	0.715	1.460	0.907	

### Methylation level of the YTH domain family

**Figure 7:**
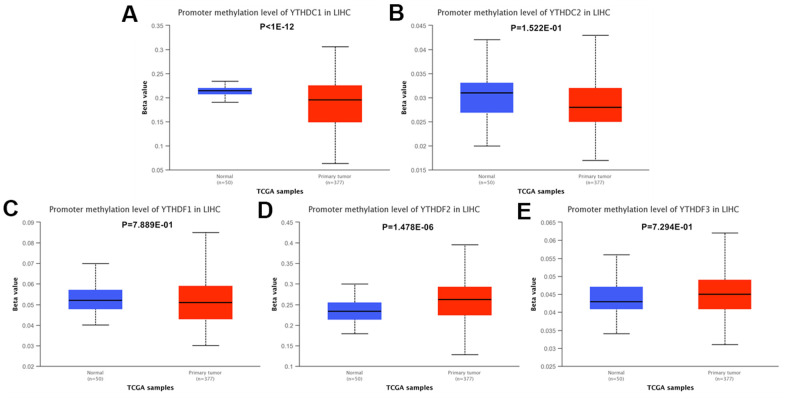
**The methylation levels of the YTH domain family in HCC tissues.** (**A**–**E**) The methylation values of (**A**) YTHDC1, (**B**) YTHDC2, (**C**) YTHDF1, (**D**) YTHDF2, (**E**) YTHDF3 were evaluated using the Ualcan database.

Furthermore, we analyzed the methylation levels of YTH domain family by searching Ualcan database. We found that the methylation levels of YTHDC1 (p < 1E-12) was higher in HCC patients than in normal cases; while YTHDF2 (p = 1.478E-06) was lower in HCC patients (Figure 7). These downregulated methylation values might explain their difference in expression levels to some extent.

## DISCUSSION

YTH domain family members were the m6A “reader” proteins which were firstly identified. M6A modification can be recognized by YTH domain family proteins in a methylation-dependent manner, which is identified by sequence comparison and is found in 174 different proteins expressed in eukaryotes [11]. There are five YTH domain-containing proteins are found in human genome, which can be classified into three categories: YTHDC1, YTHDC2 and YTHDF1–3 [12]. YTH domain family proteins are collected by m6A and linked to several mRNA metabolism including mRNA splicing, translation, nuclear export, and mRNA degradation [13, 14]. Therefore, YTH domain family proteins may be involved in many tumor physiological processes [8, 15]. In literature, YTHDF1, an oncogene in HCC, is reported to promote the epithelial mesenchymal transition (EMT) of HCC cells by accelerating the translation of snail family transcriptional repressor 1 (SNAI1) [16]. Meanwhile, Overexpression of YTHDF1 is also presented to promote HCC cell proliferation, differentiation and metastasis via accelerating the transcription of frizzled5 mRNA through an m6A-dependent manner [17]. Functioning as a tumor suppressor, high level of YTHDF2 could inhibit the proliferation and neoplasia by degrading EGFR mRNA of HCC cells in an m6A-dependent manner [18]. However, YTHDF2 also plays a repressive role in HCC by promoting the reduction of inflammatory cytokines interleukin (IL)-11 [19]. However, the detailed functions and mechanisms of YTH domain family in HCC have not been fully explored and explained.

In our study, according to the TIMER, Wanderer, GEPIA and HCCDB database, we concluded that the expression levels of YTH domain family are different between HCC and normal tissues. Especially, YTHDF1 might be more significant and valuable to be as an oncogene in development and progression of HCC. Additionally, we also performed the relationship between expression levels of YTH domain family and HCC pathological stage. We found that the expression level of YTHDC1, YTHDF1 and YTHDF2 were increased with the HCC stage progressed, suggesting that these three proteins might participate in disease progression in HCC patients. Moreover, HCC patients with high expression of YTHDF1 and YTHDF2 had a poor OS time. HCC patients with high expression of YTHDF1 had a poor PFS value. These databases implied that YTH domain family is crucial for the progression and survival of HCC patients. However, more specific studies about the expression and prognostic values of YTH domain family in patients with HCC needs to be further explored.

To better understand the potential mechanism of YTH domain family in HCC, we performed GO and KEGG pathway enrichment analysis to explore the function of YTH domain family. We found that the biological functions of YTH domain family participated primarily in the posttranslational modification pathways, such as peptidyl-serine modification and peptidyl-tyrosine modification. A previous study has shown that serine biosynthesis pathway and tyrosine relative kinase inhibitors play significant roles in the regulation of cancer progress and immunotherapy for HCC [20, 21]. In addition, the KEGG enrichment pathway analysis revealed that YTH domain family could also participate in several cancer-associated pathways, such as negative regulation of cellular component movement pathway [8, 17–19]. These findings suggest that YTH domain family could be drawn into the progression of HCC through regulating protein posttranslational modification pathways.

Immune molecules play a critical role in the regulating tumor responses [22–24]. A growing number of researches have focused on the interactions between tumors and immune cells [25, 26] and found that the expression levels of YTH domain family were strongly related to the immune cells infiltration, including B cells, CD4+ T cells, macrophages, neutrophils and dendritic cells. Our result aligned with other researches, indicated that immune cells infiltration could have important effects on the immunotherapy and clinical outcomes of cancers [27, 28]. A recent study has pointed that the m6A regulators were significantly associated with tumor immune microenvironment in HCC [29]. For example, YTHDF2 expression was correlated with some infiltrating immune cells in HCC, such as B cells, CD8+ T cells, CD4 + T cells, macrophages, neutrophils, and DCs [30]. In addition, Shi et al. [31] found that YTHDF1 facilitated NSCLC cell proliferation and xenograft tumorigenesis by regulating the translational efficiency of several immune checkpoints. In our study, we found that the expression profiles of YTHDF1 is significantly linked to the infiltration of B cells, CD8+ T cells, dendritic cells. These data indicated that the YTH domain family could regulate the infiltration of immune cells, which may trigger an intense immune response of infiltrating immune cells in the tumor environment.

In the present study, there still exist some limitations. Firstly, the statistics we analyzed were mainly from the databases, therefore, we should present more *in vitro* and *in vivo* experiments to identify the functions and mechanisms of YTH domain family in HCC. Secondly, the study did not conduct some survey concerning the therapeutic outcome of HCC patients. Thus, the outcomes need to be validated and amended in the future multicenter, large-scale clinical trials. Finally, it is important to explore prognostic effects of the YTH domain family on HCC patients in order to improve their application values in clinic.

In conclusion, we comprehensively analyzed the profiles of YTH domain family in HCC from the perspective of bioinformatics, including expression levels, prognostic values and immune response. The results showed the YTH domain family, especially YTHDF1, possessed great potentiality as therapeutic targets and prognostic biomarkers in HCC via regulating multiple mechanisms. These findings might help clinicians design effective therapeutic strategies to improve the treatment effect and prognosis of HCC patients.

## MATERIALS AND METHODS

### Wanderer

Wanderer, an intuitive Web tool, which permits real-time access and visualization of gene expression and DNA methylation profiles from TCGA using gene targeted queries [32]. Wanderer was applied to evaluate the YTH domain family expression profiles in tumor tissues and normal tissues.

### GEPIA

Gene Expression Profiling Interactive Analysis (GEPIA) is a database that is designed to help end users fully understand gene expression from a more holistic perspective [33]. We used “Single Gene Analysis” in GEPIA to evaluate the expression levels of YTH domain family members in tumor tissues compared with normal tissues. We also used this database to evaluate the effects of YTH domain family in pathological stage, prognosis and so on. The p-value was < 0.05.

### HCCDB

HCCDB is a one-stop online database to explore HCC gene expression with user-friendly interfaces [34]. The global differential gene expression landscape of HCC was built by analyzing the consistently differentially expressed genes across 15 public HCC gene expression datasets. In the study, we choose HCCDB18 to identify whether there existed significant difference of YTH domain protein expression between tumor samples and adjacent samples. We also displayed the co-expressed genes of YTH domain family in HCC samples, adjacent tissue samples, and normal liver samples, respectively.

### Kaplan-Meier plotter

Kaplan-Meier plotter is a database assessing the relationship between gene expression with the survival trend of numerous cancer patients [35]. In our study, we evaluated the prognosis with HCC patients through means of OS and PFS curves. It showed statistical significance if the p-value < 0.05.

### cBioPortal

cBioPortal provides a user-friendly analysis strategy concerning gene-disease associations on over 200 cancer cases [36]. In our study, we analyzed the co-expression profiles and genetic alterations of YTH domain family in HCC tissues by searching cBioPortal.

### STRING

STRING was applied to explore potential protein-protein interactions (PPIs). Furthermore, this database provides all resources concerning the interactive network of multiple proteins [37]. At the same time, we analyzed the YTH domain family member-associated PPI network using STRING and Cytoscape [38].

### GeneMANIA

GeneMANIA has been applied in scientific research for many years and is very convenient for identifying protein-protein interactive networks [39]. Using GeneMANIA, we successfully identified the genes associated with the YTH domain family.

### WebGestalt

WebGestalt aims to provide users with a better understanding of gene interpretation. Researchers can obtain enrichment results from this database [40]. In this study, we analyzed GO and KEGG enrichment pathways associated with the YTH domain family in HCC disease.

### TIMER

TIMER is used to evaluate the connection between infiltrating immune cells and cancer cells, which could provide more rational strategies for an improved therapeutic response and prognosis [41]. We mainly performed correlation analysis between the expression of YTH domain family with immune cells using the TIMER database.

### Ualcan

The Ualcan data portal can help to identify candidate biomarkers of specific cancer subtypes, with diagnostic, prognostic therapeutic implication [42]. In our study, we evaluated the relationship between the expression of YTH domain family and methylation levels.

## Supplementary Material

Supplementary Figure 1

Supplementary Table 1

Supplementary Table 2
