# Supplementary material for: YTH domain family: potential prognostic targets and immune-associated biomarkers in hepatocellular carcinoma
Source: Aging (Albany NY). 2021 Nov 8;13(21):24205–18. doi: 10.18632/aging.203674 (PMC8610120; doi:10.18632/aging.203674)
Supplement: Supplementary Figure 1 [file aging-13-203674-s001.pdf]

SUPPLEMENTARY FIGURE

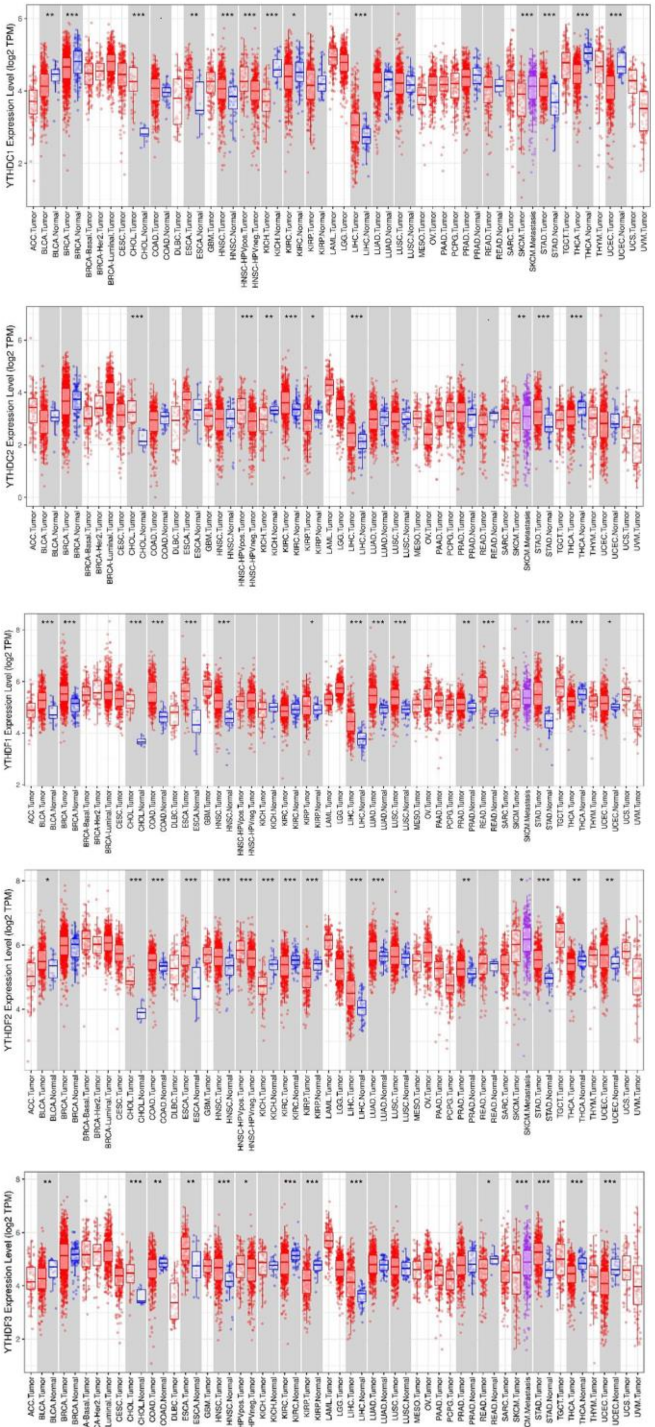

Supplementary Figure 1. YTH domain family expression in different cancer types via the tumor immune estimation resource (TIMER) database.
