# Supplementary material for: YTH domain family: potential prognostic targets and immune-associated biomarkers in hepatocellular carcinoma
Source: Aging (Albany NY). 2021 Nov 8;13(21):24205–18. doi: 10.18632/aging.203674 (PMC8610120; doi:10.18632/aging.203674)
Supplement: Supplementary Table 1 [file aging-13-203674-s002.pdf]

SUPPLEMENTARY TABLE

Supplementary Table 1. The main databases applied to evaluate the expression and functions of YTH domain family in the biological process of ovarian serous carcinoma.

Databases	Authors	Publication date	Samples	URL
Wanderer	Díez-Villanueva, A. et al.	2015	Tissues	http://maplab.imppc.org/wanderer/
GEPIA2	Tang Z. et al.	2019	Tissues	http://gepia.cancer-pku.cn/
HCCDB	Lian Q. et al.	2018	Tissues	http://lifeome.net/database/hccdb
Kaplan-Meierplotter	Gyorffy B. et al.	2005	Tissues	http://kmplot.com/analysis/
cBioPortal	Cerami E. et al.	2012	Tissues	http://www.cbioportal.org/
STRING v11	Szklarczyk D. et al.	2019	-	https://string-db.org/
GeneMANIA	Warde-Farley D. et al.	2010	-	http://genemania.org/
WebGestalt	Liao Y. et al.	2019	-	http://webgestalt.org/
Timer 2.0	Li T. et al.	2020	Tissues	https://cistrome.shinyapps.io/timer/
DiseaseMeth 2.0	Xiong Y. et al.	2017	Tissues	http://biobigdata.hrbmu.edu.cn/diseasemeth/

GEPIA, Gene expression profiling interactive analysis; WebGestalt, the web-based GEne SeT AnaLysis Toolkit.
