# Supplementary material for: YTH domain family: potential prognostic targets and immune-associated biomarkers in hepatocellular carcinoma
Source: Aging (Albany NY). 2021 Nov 8;13(21):24205–18. doi: 10.18632/aging.203674 (PMC8610120; doi:10.18632/aging.203674)
Supplement: Supplementary Table 2 [file aging-13-203674-s003.docx]

**Supplementary Table 2. The database of cBioPortal applied to search the altered genes of the YTH domain family in tissues of hepatocellular carcinoma.**

| **Gene** | **Cytoband** | **Log Ratio** | **p-Value** | **q-Value** | **expression** |
| --- | --- | --- | --- | --- | --- |
| TSC1 | 9q34.13 | 0.15 | 6.54E-04 | 0.0752 | Altered group |
| SMAD3 | 15q22.33 | 0.15 | 1.09E-03 | 0.0752 | Altered group |
| SRC | 20q11.23 | 0.25 | 1.16E-03 | 0.0752 | Altered group |
| PDK1_PS241 | 15q22.1-q22.33 | 0.14 | 1.48E-03 | 0.0752 | Altered group |
| MAPK14 | 6p21.31 | 0.13 | 2.38E-03 | 0.0781 | Altered group |
| RPS6_PS240_S244 | 5q21-q22 | -0.41 | 2.47E-03 | 0.0781 | Unaltered group |
| YWHAZ | 8q22.3 | 0.19 | 2.69E-03 | 0.0781 | Altered group |
| PDK1 | 2q31.1 | 0.12 | 3.46E-03 | 0.081 | Altered group |
| XRCC5 | 2q35 | 0.14 | 4.47E-03 | 0.081 | Altered group |
| AKT1_PS473 | 8p11.1 | -0.29 | 4.79E-03 | 0.081 | Unaltered group |
| AKT2_PS473 | 8p11.1 | -0.29 | 4.79E-03 | 0.081 | Unaltered group |
| AKT3_PS473 | 8p11.1 | -0.29 | 4.79E-03 | 0.081 | Unaltered group |
| BID | 22q11.21 | 0.11 | 5.26E-03 | 0.0818 | Altered group |
| RPS6_PS235_S236 | 5q21-q22 | -0.3 | 6.05E-03 | 0.0818 | Unaltered group |
| ESR1 | 6q25.1-q25.2 | -0.4 | 6.18E-03 | 0.0818 | Unaltered group |
| BRAF | 7q34 | 0.19 | 6.88E-03 | 0.0818 | Altered group |
| GSK3A | 19q13.2 | 0.09 | 7.60E-03 | 0.0818 | Altered group |
| GSK3B | 3q13.33 | 0.09 | 7.60E-03 | 0.0818 | Altered group |
| RAB25 | 1q22 | -0.32 | 7.66E-03 | 0.0818 | Unaltered group |
| FN1 | 2q35 | 0.27 | 8.79E-03 | 0.0892 | Altered group |
| EIF4EBP1_PS65 | 8p12 | 0.1 | 9.76E-03 | 0.0943 | Altered group |
| BCL2 | 18q21.33 | -0.11 | 0.0121 | 0.11 | Unaltered group |
| PGR | 11q22.1 | -0.07 | 0.0124 | 0.11 | Unaltered group |
| CTNNB1 | 3p22.1 | 0.25 | 0.0155 | 0.131 | Altered group |
| MTOR | 1p36.22 | 0.12 | 0.0181 | 0.138 | Altered group |
| TFRC | 3q29 | 0.25 | 0.0185 | 0.138 | Altered group |
| RPTOR | 17q25.3 | 0.1 | 0.0199 | 0.138 | Altered group |
| CDKN1B | 12p13.1 | -0.11 | 0.02 | 0.138 | Unaltered group |
| ACVRL1 | 12q13.13 | -0.05 | 0.0203 | 0.138 | Unaltered group |
| TSC2 | 16p13.3 | 0.14 | 0.0209 | 0.138 | Altered group |
| FOXO3_PS318_S321 | 5q21-q22 | -0.04 | 0.0237 | 0.138 | Unaltered group |
| EGFR_PY1068 | 7q31 | -0.16 | 0.0243 | 0.138 | Unaltered group |
| CDKN1A | 6p21.2 | -0.1 | 0.0253 | 0.138 | Unaltered group |
| TP53BP1 | 15q15.3 | 0.13 | 0.0263 | 0.138 | Altered group |
| FOXO3 | 6q21 | -0.05 | 0.0269 | 0.138 | Unaltered group |
| DIRAS3 | 1p31.3 | -0.11 | 0.0276 | 0.138 | Unaltered group |
| RAF1 | 3p25.2 | 0.07 | 0.0276 | 0.138 | Altered group |
| ERBB2_PY1248 | 6q25.1 | -0.08 | 0.0282 | 0.138 | Unaltered group |
| RPS6 | 9p22.1 | 0.22 | 0.0284 | 0.138 | Altered group |
| SMAD1 | 4q31.21 | 0.16 | 0.029 | 0.138 | Altered group |
| YAP1 | 11q22.1 | 0.08 | 0.0293 | 0.138 | Altered group |
| ATM | 11q22.3 | 0.21 | 0.0295 | 0.138 | Altered group |
| ANXA1 | 9q21.13 | -0.21 | 0.0298 | 0.138 | Unaltered group |
| PECAM1 | 17q23.3 | -0.06 | 0.0299 | 0.138 | Unaltered group |
| ARAF_PS299 | 12q24.11 | -0.03 | 0.0323 | 0.146 | Unaltered group |
| STK11 | 19p13.3 | 0.1 | 0.0345 | 0.151 | Altered group |
| RBM15 | 1p13.3 | 0.16 | 0.0367 | 0.151 | Altered group |
| INPP4B | 4q31.21 | -0.18 | 0.0369 | 0.151 | Unaltered group |
| MAPK1 | 22q11.22 | 0.13 | 0.0371 | 0.151 | Altered group |
| MSH2 | 2p21-p16.3 | -0.13 | 0.0373 | 0.151 | Unaltered group |
| LCK | 1p35.2 | 0.11 | 0.038 | 0.151 | Altered group |
| AR | Xq12 | -0.19 | 0.0402 | 0.157 | Unaltered group |
| RICTOR_PT1135 | 2q31.1 | 0.08 | 0.0415 | 0.159 | Altered group |
| AKT1_PT308 | 8p11.1 | -0.12 | 0.0452 | 0.163 | Unaltered group |
| AKT2_PT308 | 8p11.1 | -0.12 | 0.0452 | 0.163 | Unaltered group |
| AKT3_PT308 | 8p11.1 | -0.12 | 0.0452 | 0.163 | Unaltered group |
| MAPK9 | 5q35.3 | 0.1 | 0.0465 | 0.163 | Altered group |
| SMAD4 | 18q21.2 | -0.04 | 0.0471 | 0.163 | Unaltered group |
| SRSF1 | 17q22 | -0.05 | 0.0473 | 0.163 | Unaltered group |
| TP53 | 17p13.1 | -0.14 | 0.0498 | 0.169 | Unaltered group |
| FOXM1 | 12p13.33 | -0.18 | 0.0532 | 0.177 | Unaltered group |
| PEA15 | 1q23.2 | 0.12 | 0.0547 | 0.178 | Altered group |
| ACACA | 17q12 | 0.23 | 0.0555 | 0.178 | Altered group |
| PRKCD_PS664 | 12p13.1-p12 | -0.04 | 0.056 | 0.178 | Unaltered group |
| FASN | 17q25.3 | -0.34 | 0.0587 | 0.183 | Unaltered group |
| TSC2_PT1462 | 1q21 | -0.1 | 0.0612 | 0.188 | Unaltered group |
| YAP1_PS127 | 20q12-q13 | 0.17 | 0.0658 | 0.196 | Altered group |
| YWHAE | 17p13.3 | -0.03 | 0.0667 | 0.196 | Unaltered group |
| ERBB3_PY1298 | 22q13.1 | 0.07 | 0.0689 | 0.196 | Altered group |
| PREX1 | 20q13.13 | -0.1 | 0.0693 | 0.196 | Unaltered group |
| RAB11A | 15q22.31 | 0.09 | 0.0694 | 0.196 | Altered group |
| RAB11B | 19p13.2 | 0.09 | 0.0694 | 0.196 | Altered group |
| PRKAA1 | 5p13.1 | 0.07 | 0.0728 | 0.202 | Altered group |
| ERCC1 | 19q13.32 | -0.15 | 0.0759 | 0.208 | Unaltered group |
| CDKN1B_PT157 | 8p12 | 0.06 | 0.0771 | 0.209 | Altered group |
| BAX | 19q13.33 | 0.11 | 0.0808 | 0.215 | Altered group |
| BECN1 | 17q21.31 | -0.06 | 0.0817 | 0.215 | Unaltered group |
| BIRC2 | 11q22.2 | 0.07 | 0.0889 | 0.231 | Altered group |
| BAP1 | 3p21.1 | -0.14 | 0.091 | 0.234 | Unaltered group |
| ERBB2 | 17q12 | -0.12 | 0.0947 | 0.24 | Unaltered group |
| BAK1 | 6p21.31 | -0.07 | 0.101 | 0.253 | Unaltered group |
| MS4A1 | 11q12.2 | 0.09 | 0.102 | 0.253 | Altered group |
| RAD50 | 5q31.1 | 0.05 | 0.104 | 0.254 | Altered group |
| ESR1_PS118 | 11q24.2 | 0.08 | 0.108 | 0.26 | Altered group |
| CHEK1 | 11q24.2 | -0.04 | 0.117 | 0.277 | Unaltered group |
| SRC_PY416 | 9p21 | -0.1 | 0.117 | 0.277 | Unaltered group |
| XBP1 | 22q12.1\|22q12 | -0.06 | 0.119 | 0.277 | Unaltered group |
| BCL2L1 | 20q11.21 | 0.04 | 0.124 | 0.28 | Altered group |
| NF2 | 22q12.2 | 0.07 | 0.124 | 0.28 | Altered group |
| KDR | 4q12 | 0.1 | 0.126 | 0.28 | Altered group |
| CHEK2_PT68 | 1p32-p31 | 0.05 | 0.127 | 0.28 | Altered group |
| CHEK1_PS345 | 5p12 | -0.05 | 0.127 | 0.28 | Unaltered group |
| YWHAB | 20q13.12 | -0.03 | 0.128 | 0.28 | Unaltered group |
| RPS6KB1_PT389 | 6q21 | -0.17 | 0.132 | 0.285 | Unaltered group |
| CLDN7 | 17p13.1 | 0.21 | 0.133 | 0.285 | Altered group |
| AKT1S1_PT246 | 17q23.1 | -0.03 | 0.149 | 0.314 | Unaltered group |
| PDCD4 | 10q25.2 | 0.1 | 0.15 | 0.315 | Altered group |
| GSK3A_PS9 | 8p11.1 | -0.09 | 0.157 | 0.322 | Unaltered group |
| GSK3B_PS9 | 8p11.1 | -0.09 | 0.157 | 0.322 | Unaltered group |
| COL6A1 | 21q22.3 | 0.09 | 0.166 | 0.338 | Altered group |
| YBX1 | 1p34.2 | 0.06 | 0.17 | 0.341 | Altered group |
| MAPK8_PT183_Y185 | | -0.06 | 0.174 | 0.341 | Unaltered group |
| TGM2 | 20q11.23 | -0.12 | 0.174 | 0.341 | Unaltered group |
| SYK | 9q22.2 | 0.17 | 0.175 | 0.341 | Altered group |
| TIGAR | 12p13.32 | 0.17 | 0.178 | 0.344 | Altered group |
| EEF2 | 19p13.3 | 0.13 | 0.19 | 0.364 | Altered group |
| PRKAA1_PT172 | 19q13.1-q13.2 | 0.1 | 0.206 | 0.391 | Altered group |
| PRDX1 | 1p34.1 | -0.06 | 0.216 | 0.406 | Unaltered group |
| SERPINE1 | 7q22.1 | 0.19 | 0.22 | 0.41 | Altered group |
| GAPDH | 12p13.31 | 0.2 | 0.223 | 0.411 | Altered group |
| IRS1 | 2q36.3 | 0.07 | 0.227 | 0.415 | Altered group |
| EGFR | 7p11.2 | 0.06 | 0.23 | 0.416 | Altered group |
| RPS6KB1 | 17q23.1 | 0.05 | 0.231 | 0.416 | Altered group |
| BCL2L11 | 2q13 | 0.1 | 0.237 | 0.423 | Altered group |
| PIK3R1 | 5q13.1 | 0.06 | 0.245 | 0.429 | Altered group |
| PIK3R2 | 19p13.11 | 0.06 | 0.245 | 0.429 | Altered group |
| MRE11 | 11q21 | 0.03 | 0.254 | 0.441 | Altered group |
| MAP2K1_PS217_S221 | 5q21-q22 | 0.06 | 0.257 | 0.443 | Altered group |
| CDH3 | 16q22.1 | 0.04 | 0.262 | 0.448 | Altered group |
| CDH2 | 18q12.1 | 0.03 | 0.266 | 0.449 | Altered group |
| WWTR1 | 3q25.1 | 0.03 | 0.272 | 0.456 | Altered group |
| SHC1_PY317 | 13q14.2 | -0.04 | 0.279 | 0.464 | Unaltered group |
| PEA15_PS116 | 1p36.2 | 0.11 | 0.288 | 0.472 | Altered group |
| CDKN1B_PT198 | Xp11.4-p11.2 | 0.02 | 0.29 | 0.472 | Altered group |
| ERRFI1 | 1p36.23 | 0.05 | 0.29 | 0.472 | Altered group |
| CASP7 | 10q25.3 | 0.11 | 0.302 | 0.478 | Altered group |
| RPS6KA1_PT359_S363 | 5q21-q22 | -0.04 | 0.303 | 0.478 | Unaltered group |
| NRAS | 1p13.2 | -0.02 | 0.303 | 0.478 | Unaltered group |
| DIABLO | 12q24.31 | 0.09 | 0.306 | 0.478 | Altered group |
| MAPK14_PT180_Y182 | 5q21-q22 | -0.09 | 0.307 | 0.478 | Unaltered group |
| VHL | 3p25.3 | -0.23 | 0.308 | 0.478 | Unaltered group |
| YBX1_PS102 | 20q12-q13 | 0.03 | 0.324 | 0.498 | Altered group |
| MTOR_PS2448 | 8p11.1 | -0.04 | 0.334 | 0.51 | Unaltered group |
| MSH6 | 2p16.3 | 0.06 | 0.355 | 0.535 | Altered group |
| RAF1_PS338 | 1q44 | 0.02 | 0.356 | 0.535 | Altered group |
| BRCA2 | 13q13.1 | -0.03 | 0.358 | 0.535 | Unaltered group |
| STMN1 | 1p36.11 | 0.03 | 0.367 | 0.542 | Altered group |
| PXN | 12q24.23 | 0.07 | 0.37 | 0.542 | Altered group |
| CAV1 | 7q31.2 | 0.1 | 0.371 | 0.542 | Altered group |
| CCNE2 | 8q22.1 | 0.04 | 0.38 | 0.551 | Altered group |
| PRKCA_PS657 | 4q24 | -0.05 | 0.384 | 0.552 | Unaltered group |
| IGFBP2 | 2q35 | 0.13 | 0.386 | 0.552 | Altered group |
| STAT3_PY705 | 19q13.33 | -0.05 | 0.403 | 0.572 | Unaltered group |
| RAD51 | 15q15.1 | -0.03 | 0.414 | 0.579 | Unaltered group |
| RPS6KA1 | 1p36.11 | -0.04 | 0.415 | 0.579 | Unaltered group |
| MAP2K1 | 15q22.31 | 0.03 | 0.416 | 0.579 | Altered group |
| PIK3CA | 3q26.32 | -0.02 | 0.424 | 0.585 | Unaltered group |
| KIT | 4q12 | 0.05 | 0.437 | 0.6 | Altered group |
| NOTCH1 | 9q34.3 | -0.03 | 0.45 | 0.614 | Unaltered group |
| RICTOR | 5p13.1 | 0.09 | 0.467 | 0.63 | Altered group |
| CCNB1 | 5q13.2 | 0.06 | 0.472 | 0.63 | Altered group |
| ANXA7 | 10q22.2 | 0.02 | 0.477 | 0.63 | Altered group |
| AKT1 | 14q32.33 | -0.05 | 0.482 | 0.63 | Unaltered group |
| AKT2 | 19q13.2 | -0.05 | 0.482 | 0.63 | Unaltered group |
| AKT3 | 1q43-q44 | -0.05 | 0.482 | 0.63 | Unaltered group |
| EEF2K | 16p12.2 | -0.05 | 0.485 | 0.63 | Unaltered group |
| DVL3 | 3q27.1 | 0.03 | 0.487 | 0.63 | Altered group |
| ERBB3 | 12q13.2 | 0.04 | 0.495 | 0.636 | Altered group |
| NDRG1_PT346 | 22q11.21 | -0.07 | 0.516 | 0.659 | Unaltered group |
| ITGA2 | 5q11.2 | -0.03 | 0.521 | 0.661 | Unaltered group |
| STAT5A | 17q21.2 | 0.06 | 0.525 | 0.663 | Altered group |
| NRG1 | 8p12 | -0.02 | 0.542 | 0.678 | Unaltered group |
| MAPK1_PT202_Y204 | 5q21-q22 | -0.07 | 0.551 | 0.678 | Unaltered group |
| MAPK3_PT202_Y204 | 5q21-q22 | -0.07 | 0.551 | 0.678 | Unaltered group |
| EGFR_PY1173 | 3p25 | 0.01 | 0.551 | 0.678 | Altered group |
| CDH1 | 16q22.1 | -0.07 | 0.556 | 0.68 | Unaltered group |
| JUN_PS73 | 17q21.31 | -0.03 | 0.567 | 0.689 | Unaltered group |
| EIF4EBP1_PT70 | 17q21 | 0.02 | 0.574 | 0.693 | Altered group |
| EIF4G1 | 3q27.1 | 0.04 | 0.588 | 0.706 | Altered group |
| GAB2 | 11q14.1 | 0.03 | 0.6 | 0.717 | Altered group |
| SCD | 10q24.31 | -0.02 | 0.624 | 0.74 | Unaltered group |
| PRKCA | 17q24.2 | 0.03 | 0.63 | 0.744 | Altered group |
| CHEK2 | 22q12.1 | -0.03 | 0.649 | 0.762 | Unaltered group |
| EIF4EBP1 | 8p11.23 | 0.02 | 0.667 | 0.775 | Altered group |
| EIF4E | 4q23 | -0.01 | 0.668 | 0.775 | Unaltered group |
| CCND1 | 11q13.3 | -0.01 | 0.68 | 0.785 | Unaltered group |
| MYC | 8q24.21 | -0.02 | 0.704 | 0.807 | Unaltered group |
| RB1_PS807_S811 | 5q21-q22 | -0.03 | 0.708 | 0.807 | Unaltered group |
| EIF4EBP1_PT37 | 8p12 | -0.03 | 0.718 | 0.814 | Unaltered group |
| GATA3 | 10p14 | -0.01 | 0.722 | 0.815 | Unaltered group |
| G6PD | Xq28 | 0.02 | 0.73 | 0.819 | Altered group |
| NFKB1_PS536 | 16p11.2 | 0.03 | 0.739 | 0.821 | Altered group |
| MET_PY1235 | 11q13 | -0.01 | 0.74 | 0.821 | Unaltered group |
| PARK7 | 1p36.23 | 0.01 | 0.746 | 0.823 | Altered group |
| CDK1 | 10q21.2 | -0.01 | 0.755 | 0.828 | Unaltered group |
| SRC_PY527 | 9p21 | -0.02 | 0.786 | 0.855 | Unaltered group |
| MYH11 | 16p13.11 | -0.09 | 0.788 | 0.855 | Unaltered group |
| XRCC1 | 19q13.31 | 0.01 | 0.819 | 0.882 | Altered group |
| BAD_PS112 | 19q13.1-q13.2 | -0.01 | 0.822 | 0.882 | Unaltered group |
| GSK3A_PS21_S9 | 5q21-q22 | 0.01 | 0.846 | 0.899 | Altered group |
| GSK3B_PS21_S9 | 5q21-q22 | 0.01 | 0.846 | 0.899 | Altered group |
| SQSTM1 | 5q35.3 | 0.03 | 0.852 | 0.901 | Altered group |
| CCNE1 | 19q12 | -0.03 | 0.87 | 0.915 | Unaltered group |
| ACACA_PS79 | 3q13.3 | 0.01 | 0.881 | 0.917 | Altered group |
| ACACB_PS79 | 8p11.1 | 0.01 | 0.881 | 0.917 | Altered group |
| MYH9_PS1943 | 10q11.22 | -0.01 | 0.89 | 0.922 | Unaltered group |
| PRKCB_PS660 | 5q21-q22 | -0.01 | 0.9 | 0.926 | Unaltered group |
| HSPA1A | 6p21.33 | 0.01 | 0.904 | 0.926 | Altered group |
| TUBA1B | 12q13.12 | -0.01 | 0.911 | 0.928 | Unaltered group |
| ASNS | 7q21.3 | 0.01 | 0.914 | 0.928 | Altered group |
| ETS1 | 11q24.3 | 0.01 | 0.923 | 0.933 | Altered group |
| PCNA | 20p12.3 | 0 | 0.947 | 0.951 | Unaltered group |
| PTEN | 10q23.31 | 0 | 0.974 | 0.974 | Altered group |
